# Surface characteristics and in vitro biocompatibility of titanium preserved in a vitamin C-containing saline storage solution

**DOI:** 10.1007/s10856-023-06769-5

**Published:** 2024-01-11

**Authors:** Wen-si Zhang, Yao Liu, Shui-Yi Shao, Chang-qing Shu, Yi-heng Zhou, Song-mei Zhang, Jing Qiu

**Affiliations:** 1 Department of Oral Implantology, Affiliated Stomatological Hospital of Nanjing Medical University, Nanjing, PR China; 2 Jiangsu Province Key Laboratory of Oral Diseases, Nanjing, PR China; 3 Department of Comprehensive Care, Tufts University School of Dental Medicine Boston, Boston, MA USA; 4 Jiangsu Province Engineering Research Center of Stomatological Translational Medicine, Nanjing, PR China

## Abstract

The purpose of this study is to explore a storage solution for titanium implants and investigate its osteogenic properties. The commercial pure titanium (cp-Ti) surface and double-etched (SLA) titanium surface specimens were preserved in air, saline, 10 mM Vitamin C (VitC)-containing saline and 100 mM VitC-containing saline storage solutions for 2 weeks. The surface microtopography of titanium was observed by scanning electron microscopy (SEM), the surface elemental compositions of the specimens were analyzed by Raman and X-ray photoelectron spectroscopy (XPS), and water contact angle and surface roughness of the specimens were tested. The protein adsorption capacity of two titanium surfaces after storage in different media was examined by BCA kit. The MC3T3-E1 osteoblasts were cultured on two titanium surfaces after storage in different media, and the proliferation, adhesion and osteogenic differentiation activity of osteoblasts were detected by CCK-8, laser confocal microscope (CLSM) and Western blot. The SEM results indicated that the titanium surfaces of the air group were relatively clean while scattered sodium chloride or VitC crystals were seen on the titanium surfaces of the other three groups. There were no significant differences in the micromorphology of the titanium surfaces among the four groups. Raman spectroscopy detected VitC crystals on the titanium surfaces of two experimental groups. The XPS, water contact angle and surface roughness results suggested that cp-Ti and SLA-Ti stored in 0.9% NaCl and two VitC-containing saline storage solutions possessed less carbon contamination and higher surface hydrophilicity. Moreover, the protein adsorption potentials of cp-Ti and SLA-Ti surfaces were significantly improved under preservation in two VitC-containing saline storage solutions. The results of in vitro study showed that the preservation of two titanium surfaces in 100 mM VitC-containing saline storage solution upregulated the cell adhesion, proliferation, osteogenic related protein expressions of MC3T3-E1 osteoblasts. In conclusion, preservation of cp-Ti and SLA-Ti in 100 mM VitC-containing saline storage solution could effectively reduce carbon contamination and enhance surface hydrophilicity, which was conducive to osteogenic differentiation of osteoblasts.

**Graphical Abstract:**

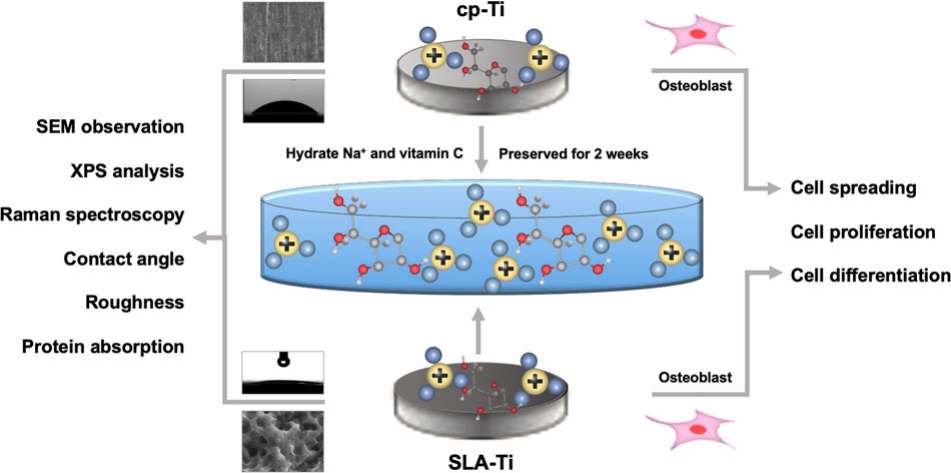

## Introduction

Pure titanium exhibits excellent biocompatibility and corrosion resistance, titanium implants are widely used as implant restorations for dentition defects and hypodontia. Currently, various clinical techniques, such as large particle sandblasting acid etching (SLA), large particle sandblasting acid etching, and micro arc oxidation, are used to construct a surface micrometer scale rough topography, in order to promote osteoblast adhesion, proliferation and differentiation to increase the contact area and mechanical chimerism of the implant with bone [1–3]. However, recent studies have revealed that the surface activity of titanium implants is not stable, both pure and modified titanium surfaces undergo a time-dependent bioactive aging process that leads to osteoconductive degradation. Att et al. showed that the biological aging phenomenon on the titanium surface was related to the decrease capacity of protein adsorption and osteogenic cell adhesion, which cause the progressive accumulation of hydrocarbons on the titanium surface under ambient conditions [4]. The carbon content on the titanium surface also increases with prolonged preparation time. However, an aging-like time-dependent biological degradation of titanium surfaces from bioactive to bioinert and the osteoblast potential ability of migration, attachment, spread, proliferation and differentiation decreased observably on old titanium surfaces in an age-dependent manner. Lu et al. and Hayashi et al. studies confirmed that air-exposed titanium surfaces adsorb airborne hydrocarbons, and the resulting accumulation of carbon can further inhibit osteoblastic behavior on titanium surfaces [5, 6]. Therefore, this phenomenon implies that the activation and bioactivity of titanium surface are influenced by the storage method, which can protect it from time-dependent degradation. It has been found that preserving titanium implants in isotonic saline, distilled water or vacuum packaging can effectively preserve hydrophilicity immediately after fabrication.

There are various indications that preserving the implant in a liquid is a simple, effective and economical anti-aging strategy [7, 8]. Research has shown that therea are no significant differences in protein adsorption and apatite formation capacity between freshly prepared alkali-heated titanium surfaces and titanium surfaces stored in vacuum for 52 weeks [9]. In our previous study, we observed varying degrees of carbon accumulation on the pure titanium surface after preservation for a 2-week period. Furthermore, we found that the hydrophilicity of the pure titanium surface was positively correlated with the concentration of NaCl solution. Notably, storing the pure titanium surface in a high concentration of NaCl solution significantly reduced carbon contamination. This suggests that the concentration of salt storage solution can influence carbon accumulation on the titanium surface, providing the implant with the a unique advantage of resistance to aging when stored in a saline environment.

In the human body, Vitamin C (VitC) acts as a highly effective antioxidant, capable of reducing the oxidative stress caused by ascorbate peroxidase and scavenging various reactive oxygen species (ROS). It has been extensively studied for its potential in treating different bone diseases. Research has demonstrated that VitC can reduce bone resorption by counteracting oxidative stress induced by ROS [10]. Additionally, VitC inhibits the bone resorption process by downregulating the expression of RANKL [11–13]. In humans, VitC is widely recognized as a potent antioxidant that helps alleviate oxidative stress caused by ascorbate peroxidase [14–17]. Modified titanium, despite being based on titanium metal, undergoes changes in its bioactivity and physicochemical properties, including surface morphology, hydrophilicity, surface charge, roughness, and chemical composition [18]. Therefore, in this study, water-soluble VitC was dissolved in 0.9% NaCl to dissociate H^+^ from the enolic hydroxyl group of VitC molecules. This resulted in the formation of a hydrated ionic layer that adsorbs on the titanium surface, aiming to achieve antioxidant effects in vivo to maintain titanium surface activity. However, the impact of this process on the composition of ionic groups on the titanium surface has not been studied and elucidated.

This experiment was designed to use VitC-containing saline as a storage medium to study the effect of active storage solution on the physicochemical properties and biological activity of different titanium surfaces.

## Materials and methods

### Specimen preparation

Preparation of VitC-containing saline storage solution: VitC powders (Sigma, USA) were prepared as 10 mM and 100 mM VitC-containing saline by dissolving in 0.9% NaCl.

Preparation of pure titanium surface (cp-Ti): commercially pure grade-2 titanium disks (99.5 wt% purity, Shanghai, China) were cut into titanium disks with diameters of 30 mm and 5 mm and thicknesses of 1 mm. All titanium disks were polished from ^#^400, ^#^600, ^#^800, ^#^1000, ^#^1200 to ^#^1500 for waterproof silicon carbide paper, followed by ultra-sonically cleaning with ethanol and double distilled water.

Acid etching titanium surface (SLA-Ti): The SLA-Ti surfaces were obtained after being sandblasted and etched with solution containing HF/HNO_3_ (room temperature, 10 min) and solution containing H_2_SO_4_ /HCl (80 °C in a water bath, 30 min), ultra-sonically cleaning with ethanol and double distilled water.

These titanium disks were stored in the air, 0.9% NaCl, 10 mM VitC-containing saline and 100 mM VitC-containing saline under ambient temperatures for 2 weeks. The cause of storage for 2 weeks seems to be a critical time period for degrading the wettability and cytocompatibility of titanium surfaces according to the results reported by Att et al. [4].

### Surface characterization

The cp-Ti and SLA-Ti samples were preserved in four different media for two weeks. They were observed using a scanning electron microscope (SEM, MAIA3 RISE, TESCAN, Czech) with an accelerating voltage of 10.0 kV and a working distance of 5 mm. Raman spectral data were recorded using a Tescan-WITec RISE microscope (TESCAN, Czech) equipped with a WITec Confocal Raman Imaging System (WITec, Ulm, Germany) The system utilized a 532 nm excitation of an Nd:YAG laser and a 20X, NA = 20, objective lens. The integration time of Raman spectra was 10 s. The roughness of samples was examined by using an optical profilometer (MicroXamTM, Phase-Shift, UP, Rtec co, United States). The surface elemental compositions were detected by the X-ray photoelectron spectroscopy (XPS, Thermo Scientific Escalab 250Xi, United States) under vacuum conditions. The wettability of samples under different storage conditions was tested by using Automatic Contact Angle Meter Model (SL200B, Kono, United States) using the liquid drop method. A droplet of 2 μL of sessile distilled water was dropped onto the surface in an ambient environment and the contact angles were measured using a standard optical contact angle meter. An optical profilometer (MicroXamTM, Phase-Shift, UP, Rtec co, USA) was used to evaluate the roughness of each sample over a 100 µm × 100 µm area. All tests were conducted in triplicate.

### Hydrophilicity assay

The contact angles were determined by a contact angle goniometer using standard optical contact Angle meter (SL200B, KONO, USA), which was measured in an ambient environment dropping 2 μl of distilled water droplets onto the specimens.

### Protein adsorption

Samples were placed in 96 well plates and tightly preserved under different conditions for 2 weeks. Except for the air group, the storage solution was discarded and the samples were rinsed with PBS three times. 2 mL α-MEM (MEM alpha; Gibco, CA, USA) containing 10% fetal bovine serum (FBS, Gibco) was added onto all the samples and incubated for 24 h. Afterward, each sample was rinsed with PBS three times again. The protein samples were harvested by lysis in RIPA buffer and BCA protein assay kit (KeyGEN BioTECH, China) was used to determine the concentration of the proteins.

### Cell culture

The MC3T3-E1 cells, which are an osteoblast-like cell line, were used as an in vitro model for studying osteoblast development [19]. The frozen MC3T3-E1 cells (Cell Bank of Chinese Academy of Sciences, Shanghai, China) were resuscitated and cultured in α-Minimum Essential Medium (α-MEM, Gibco, USA) containing 10% fetal bovine serum (FBS, Gibco, USA) and 1% penicillin/streptomycin (Gibco, USA). The cells were cultured in a humidified atmosphere of 5% CO_2_ and 95% air at 37°C. The medium was changed every two days. Cells were passaged at a ratio of 1:4 every 3–4 days when covering 80% area of the cell culture bottle. These studies were conducted under the approval of The Ethics Committee of Jiangsu Stomatological Hospital.

### Cell adhesion assay

To analyze the morphology of cells cultured on samples MC3T3-E1 cells (5 × 10^3^ cells/well) were cultured on cp-Ti and SLA-Ti samples in 96-well plates. After being cultured for 4 h, each sample was rinsed with PBS, and then fixed using 4% paraformaldehyde in PBS at room temperature for 10 min. Afterwards, each sample was stained using 100 nM Rhodamine Phalloidin (Cytoskeleton, USA) at room temperature in the dark for 30 min and then 100 nM 4′, 6′ -diamidino-2-phenylindole (Beyotime, Shanghai, China) for 2 min. The cell adhension and spreading morphology were imaged with three randomly selected fields on each sample under a laser scanning confocal microscope (LSM710, Zeiss, GER) at 200× magnifcation.

### Cell proliferation assay

The MC3T3-E1 cells (5 × 10^3^ cells per well) were cultured on the surface of specimens preserved under different storage conditions in 96 well plates. After culturing the cells for 1, 3, and 6 days, the culture medium were replaced by fresh medium containing 10% CCK-8 (Beyotime, Shanghai, China) reagent and continued to incubate for 2 h. The absorbances of the incubated media were measured by a microplate reader (Spectramax190, MD, USA) at 450 nm wavelength.

### Western blotting

After MC3T3-E1 cells (2 × 10^5^ cells per well) were cultured on cp-Ti and SLA-Ti samples for 7 days, cells were rinsed with cold PBS and then harvested by lysis in RIPA buffer. Total proteins were determined using a BCA protein assay kit (Key-GEN BioTECH, Nanjing, China). Protein extract samples (20 mg) were separated using SDS-PAGE, and then transferred to poly-vinylidene fluoride (PVDF, Millipore, MA, USA) membranes (Millipore, USA). After blocking with 5% skim milk for 1 h, the membranes were incubated with different primary antibodies against Runx2 (12556, CST, USA), OSX (ab209484, Abcam, USA), OCN(ab10911, EMD Millipore, USA), and GAPDH (60004, Proteintech, USA) overnight at 4 °C. These membranes were then incubated for 2 h with secondary antibodies (ZB-2301, Goat anti-Rabbit IgG, ZSGB-BIO, China; AP124P, Goat anti-Mouse IgG, Millipore, USA). Immobilon western chemiluminescent HRP substrate (Millipore, USA) was used to detect the protein signals. The protein expressions were determined relative to those of GAPDH that was used as an internal control.

### Statistical analysis

All results were statistically analyzed using SPSS 22.0 (SPSS Inc, Chicago, IL, USA) via one-way analysis of variance and Student-Newman-Keuls post hoc test. Significant differences were indicated as *P < 0.05.

## Results

### Surface structure and elements analysis

**Fig. 1:**
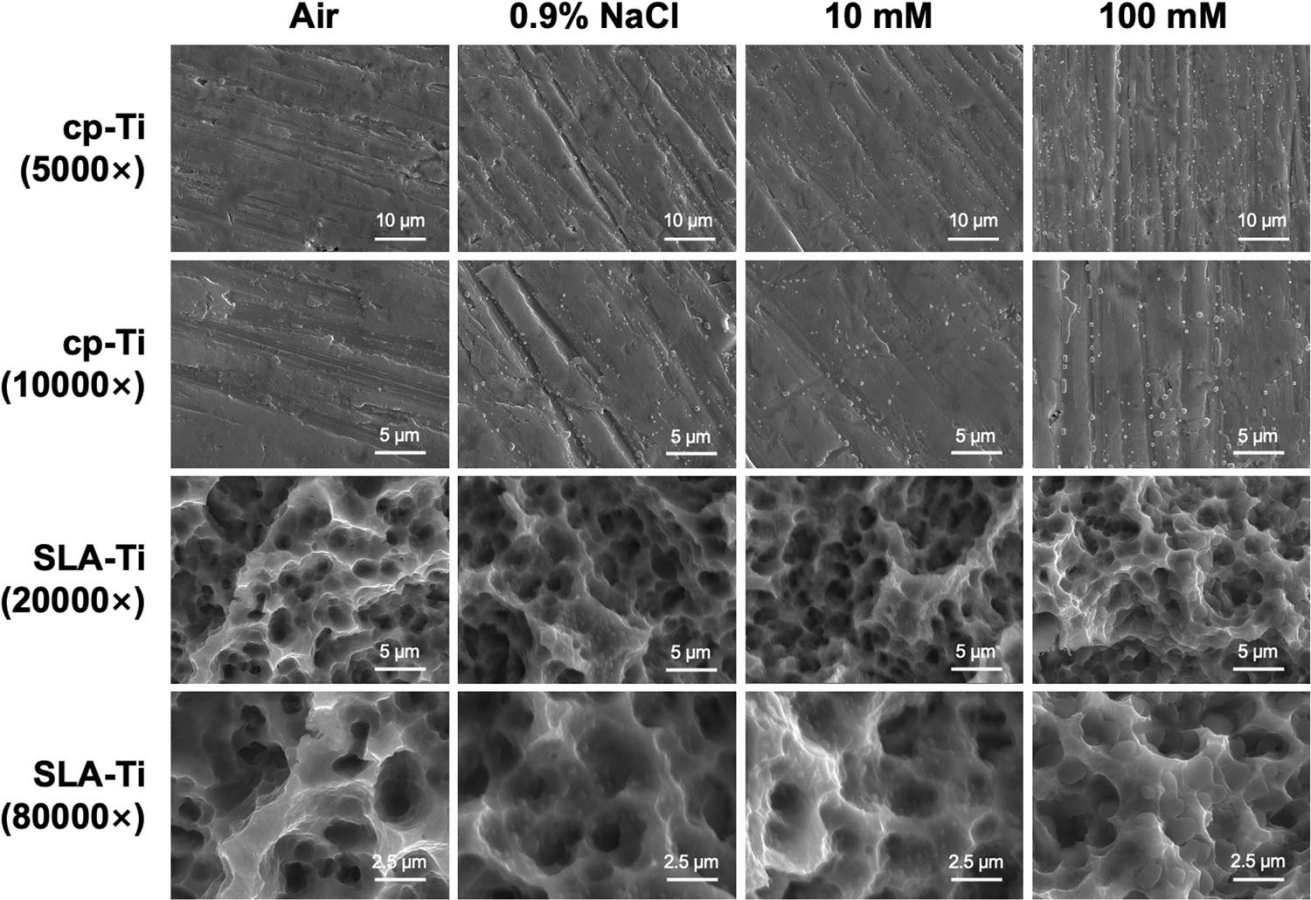
Scanning electron microscopy images of surface morphology of different surfaces after being preserved in different storage conditions for 2 weeks

The SEM images of cp-Ti and SLA-Ti samples were taken after being preserved in two different conditions for 2 weeks are shown in Fig. 1 after storage in four different media for 2 weeks. At lower magnification, there was no obvious difference within the groups of cp-Ti and SLA-Ti titanium surfaces storage in air. However, sodium chloride crystals and VitC crystals could be observed on the surfaces of both cp-Ti and SLA-Ti after storage in 0.9% NaCl, 10 mM and 100 mM VitC-containing saline storage solution. At higher magnification, the neat mechanical scratch of cp-Ti, round micro-pits with sharp edges were observed on the SLA-Ti surface (Fig. 1), and the nanosheet structures on the SLA-Ti surfaces became overlapped and entangled, forming interconnected networks. After preservation by different media, the characteristic micro topographies of the cp-Ti and SLA-Ti samples did not change.

**Fig. 2:**
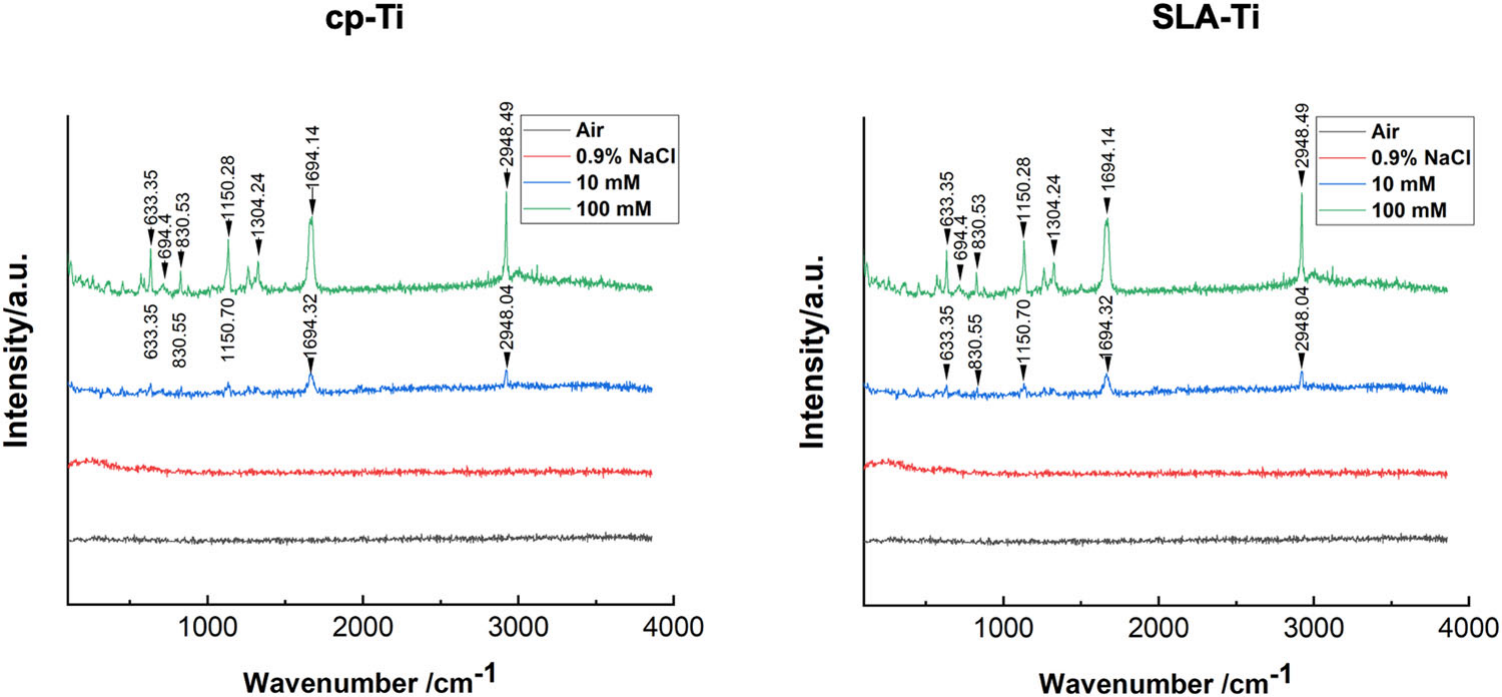
Raman analysis of cp-Ti and SLA-Ti after being preserved in different storage conditions for 2 weeks

Two titanium surfaces were preserved in different conditions for 2 weeks, the raman analysis results are shown in Fig. 2. The results demonstrated that characteristic peaks of VitC were detected on both the cp-Ti and SLA-Ti surfaces after storage in a 100 mM VitC-containing saline storage solution. These peaks correspond to the VitC molecular vibration maps. Sharps peaks at 633 cm^−1^ were observed due to the C-C ring stretching vibration of VitC, a peak around 694 cm^−1^ due to the external change of the O-H bond plane and the C-C ring stretching vibration, and 830 cm^−1^.The peak near 1304 cm^−1^ was the peak due to out of plane bending of the O-H bond, the peak around 1150 cm^−1^ was the peak due to the C-O ring stretching vibration, the peak around 1304 cm^−1^ was the peak due to the bending vibration of the O-H bond, and the peak around 1694 cm^−1^ was the peak due to the stretching vibration of the C-C double bond. The peaks of these characteristic vibrations are the responses of the structural features of VitC. In addition, the peak around 2948 cm^−1^ in the figure is the peak that would occur for organic matter in general. The characteristic peaks of VitC crystals on the surface of cp-Ti and SLA-Ti began to become indistinct after 2 weeks preserved in 10 mM VitC-containing saline storage solution due to the reduced concentration of VitC-containing saline. But the characteristic peak of VitC on SLA titanium surface after 2 weeks storage in 10 mM VitC-containing saline storage solution was more obvious than that on smooth titanium surface, which may be related to the nanostructure possessed by SLA titanium surface and the ability to adsorb more NaCl crystals and VitC crystals. While air group and 0.9% NaCl group did not detect any characteristic peaks.

**Fig. 3:**
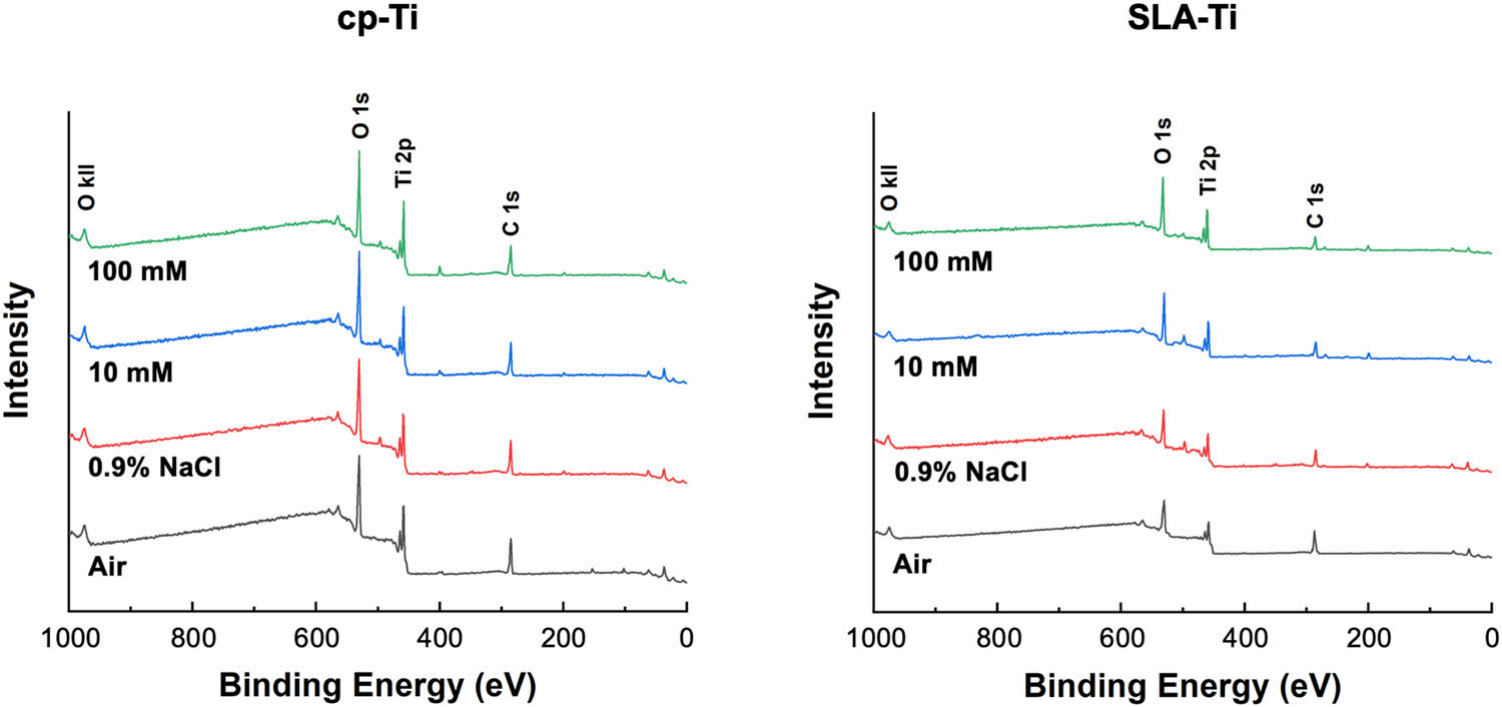
XPS spectra of cp-Ti and SLA-Ti after being preserved in different storage conditions for 2 weeks

**Fig. 4:**
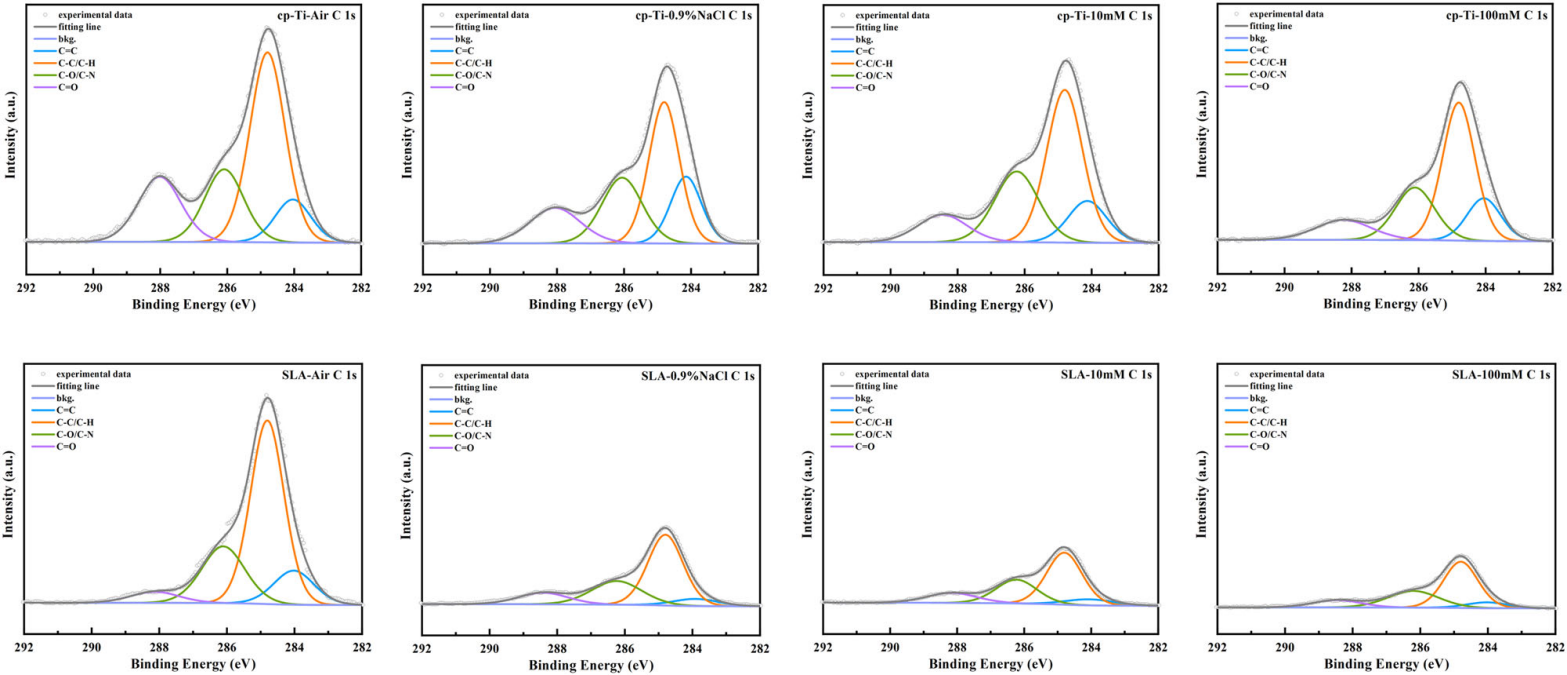
Close-up view of the high-resolution C 1s XPS spectra

The XPS analysis indicated the main chemical element on cp-Ti and SLA-Ti under different storage conditions (Fig. 3). Tables 1 and 2 display the quantitative atomic concentrations of cp-Ti and SLA-Ti under different storage conditions. Table 1 and Table 2 showed the wide scan spectrum of different samples. The quantified atomic ratios of Ti/C and O/C were given to evaluate carbon amounts between different groups. The results indicated that titanium surfaces stored in air had the highest carbon content. Titanium preserved in 100 mM VitC-containing saline suffered the lowest carbon contamination. The carbon levels of titanium disks soaked in 10 mM and 100 mM VitC-containing saline decreased gradually. In Fig. 4, the C 1 s high-resolution spectra exhibited three peaks at about 284 eV, 286 eV, and 288 eV, respectively. Carbon contamination on titanium surface is adsorbed on titanium surface with different Chemical bond, includes C-C/C-H, C-O and C=O bond. Close examination of the peaks revealed that C-C/C-H peak of air surfaces at about 284 eV ascribed to the presence of carbon-containing compounds (Fig. 4), and the 100 mM group lower than air, 0.9% NaCl and 10 mM group.

**Table 1:** Atomic composition of cp-Ti surface detected by XPS

Groups	Atomic Composition (%)			Atomic Ratio	
	C	Ti	O	O/C	Ti/C
Air	37.09	19.69	43.22	1.16	0.53
0.9% NaCl	33.96	18.43	42.57	1.25	0.54
10 mM	33.73	18.75	44.04	1.31	0.56
100 mM	32.17	20.20	45.65	1.41	0.63

**Table 2:** Atomic composition of SLA-Ti surface detected by XPS

Groups	Atomic Composition (%)			Atomic Ratio	
	C	Ti	O	O/C	Ti/C
Air	41.26	16.43	42.31	1.02	0.39
0.9% NaCl	35.13	17.86	39.53	1.13	0.51
10 mM	33.63	18.01	43.07	1.28	0.54
100 mM	29.11	21.60	45.45	1.56	0.74

**Fig. 5:**
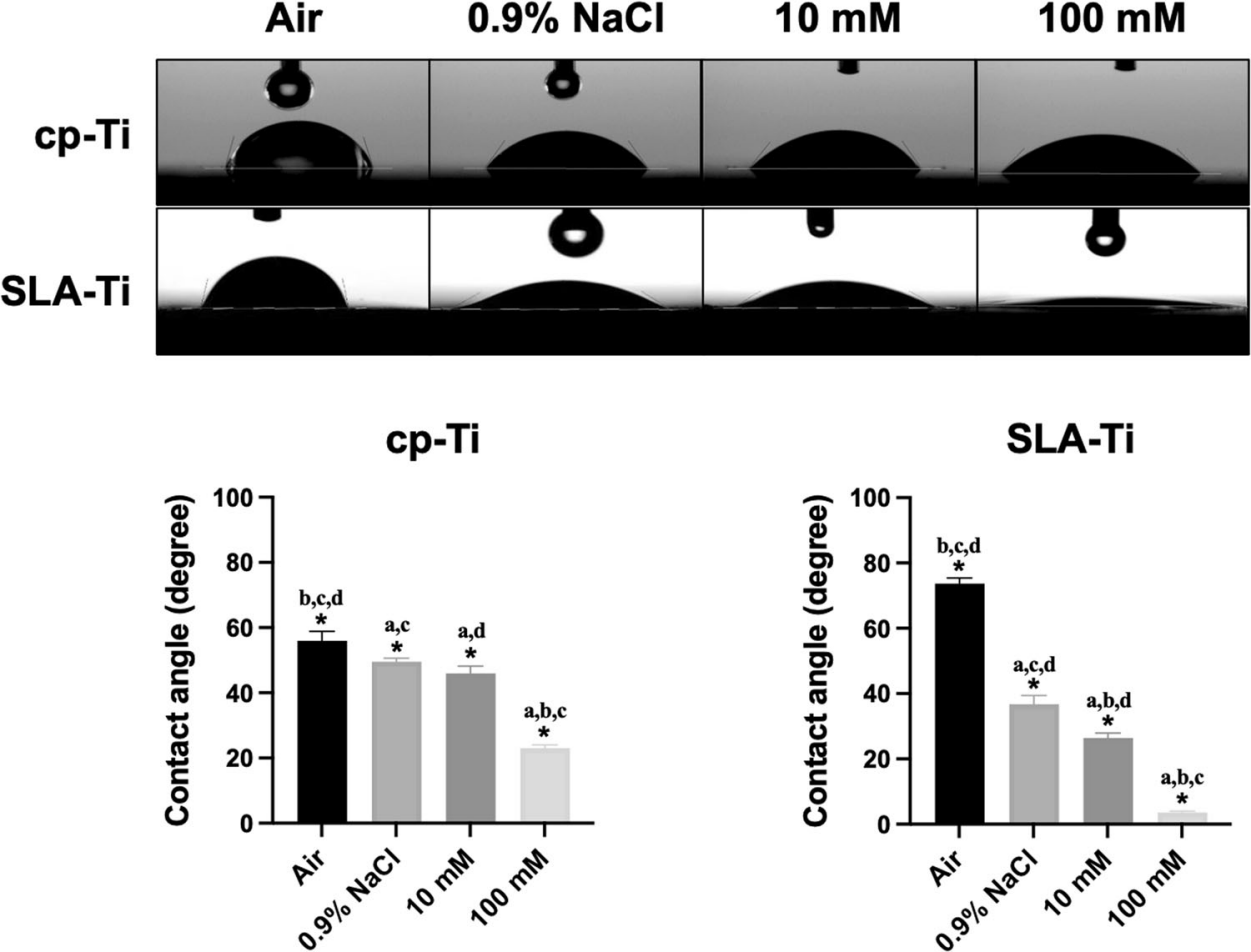
Contact angles of cp-Ti and SLA-Ti after being preserved in different storage conditions for 2 weeks. *P < 0.05. ^a^*P* < 0.05 vs. Air; ^b^*P* < 0.05 vs. 0.9%NaCl; ^c^*P* < 0.05 vs.10 mM; ^d^*P* < 0.05 vs. 100 mM

**Fig. 6:**
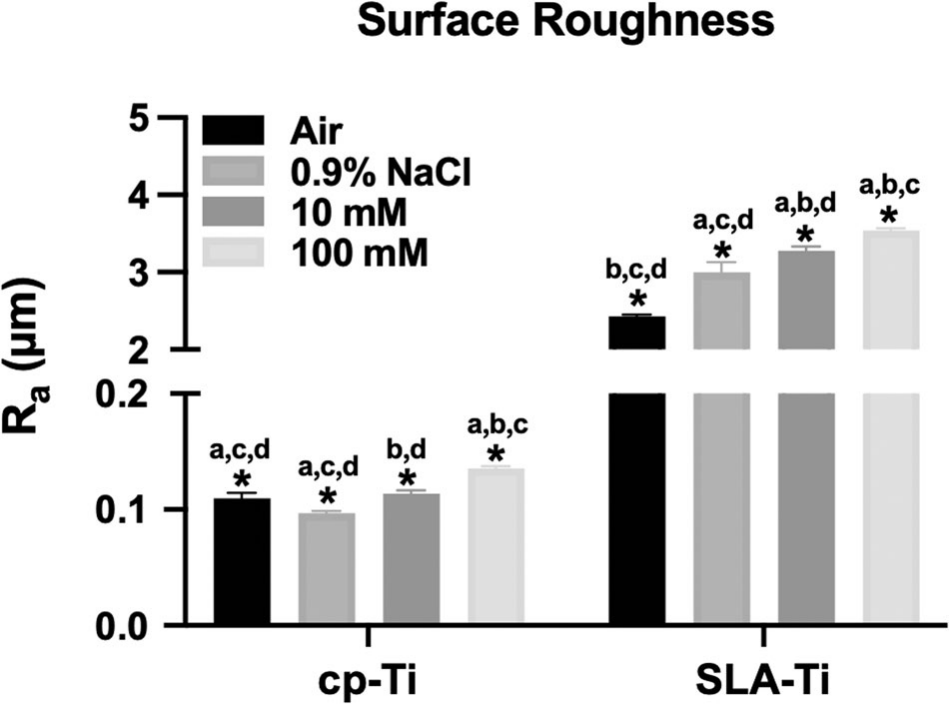
Surface roughness of cp-Ti and SLA -Ti after being preserved in different storage conditions for 2 weeks. *P < 0.05. ^a^*P* < 0.05 vs. Air; ^b^*P* < 0.05 vs. 0.9%NaCl; ^c^*P* < 0.05 vs.10 mM; ^d^*P* < 0.05 vs. 100 mM

As shown in Fig. 5, under the same storage conditions, SLA-Ti showed greater hydrophilicity than cp-Ti. And the hydrophilicity of two titanium surfaces stored in liquid was significantly higher than those stored exposed to air. Moreover, the hydrophilicity of the titanium surfaces stored in 0.9% NaCl solutions was significantly higher than that of the control groups. Notably, the titanium surfaces stored in 100 mM VitC-containing saline exhibited the highest hydrophilicity. The roughness of SLA-Ti and was significantly higher than that of cp-Ti, as shown in Fig. 6. In the two groups of specimens, the roughness of air groups increased relative to 0.9% NaCl groups, and the roughness of titanium surface increased with the increase of VitC concentration.

### Protein adsorption

**Fig. 7:**
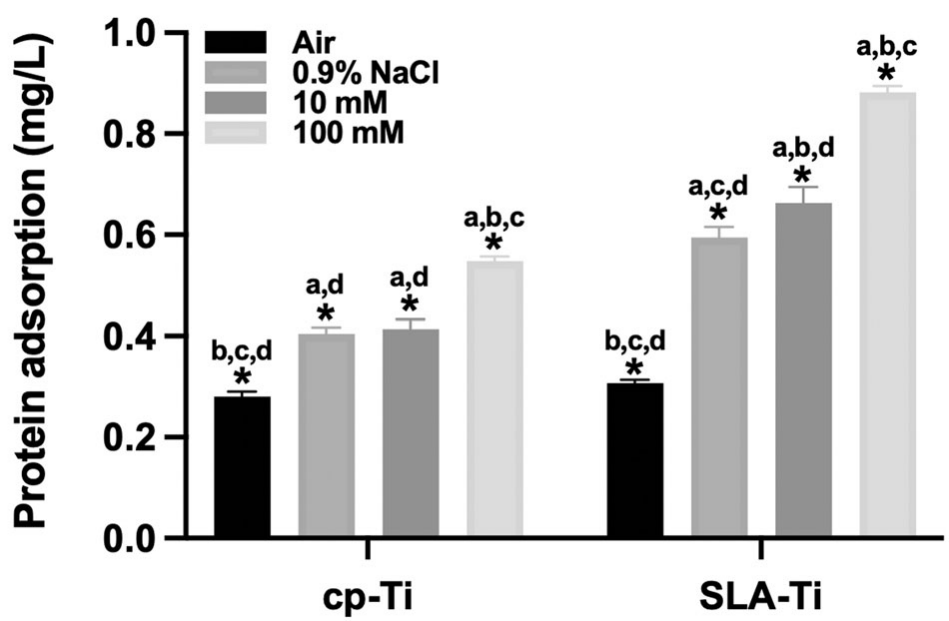
Protein adsorption of cp-Ti and SLA-Ti after being preserved in different storage conditions for 2 weeks. *P < 0.05. ^a^*P* < 0.05 vs. Air; ^b^*P* < 0.05 vs. 0.9%NaCl; ^c^*P* < 0.05 vs. 10 mM; ^d^*P* < 0.05 vs. 100 mM

The protein adsorption potential was evaluated by the amount of protein adsorbed by the samples immersed in α-MEM medium. The results of protein adsorption for cp-Ti and SLA-Ti after preserved in different conditions for 2 weeks were shown in Fig. 7. Under the same storage condition, SLA-Ti tended to adsorb more protein in the culture medium, followed by cp-Ti. For the same titanium surface, 100 mM group compared with air group, the 0.9% NaCl group and 10 mM group had higher protein adsorption capacity.

### Cell adhesion and spreading

**Fig. 8:**
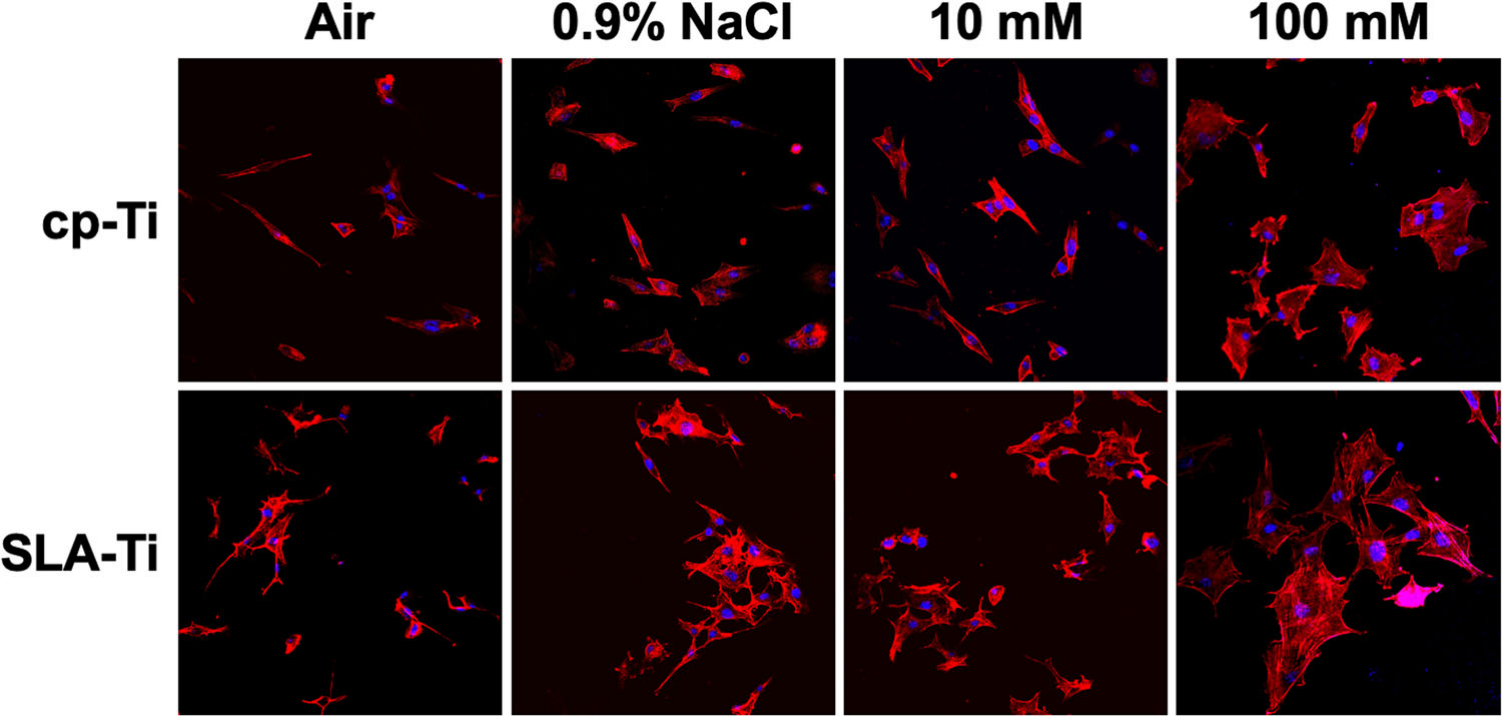
Adhesion abilities of MC3T3-E1 cells were analyzed by counting the stained nuclei with DAPI after 1 hour of incubation using a confocal laser scanning microscope

The adhesion morphologies of MC3T3-E1 cells cultured on the surfaces of samples preserved in different conditions for 2 weeks were shown in Fig. 8. Compared with air and 0.9% NaCl group, the cp-Ti and SLA-Ti osteoblast groups preserved in 100 mM VitC-containing saline storage solution exhibited adequate extension of cell pseudopods, and filamentous cytoskeleton was observed intracytoplasmically. This indicated that the samples preserved in 100 mM VitC-containing saline storage solution favored osteoblast adhesion.

### Cell proliferation

**Fig. 9:**
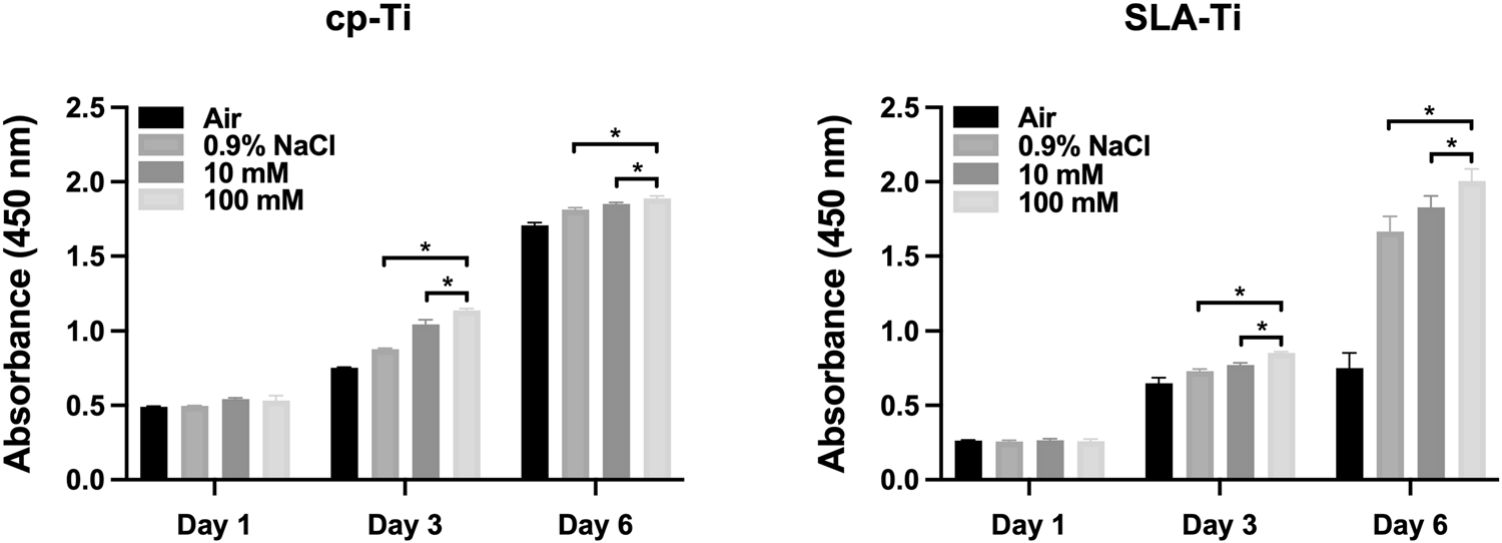
Proliferation of MC3T3-E1 cells cultured for 1, 3, and 6 days on two surfaces analyzed by CCK-8 assay. *P < 0.05

The proliferation morphologies of MC3T3-E1 cells cultured on the surfaces of samples preserved in different conditions for 2 weeks, as measured by the CCK-8 assay, were shown in Fig. 9. There was no significant difference in the proliferative activity of cells on the surface of cp-Ti and SLA-Ti after culturing for 1 day. After culturing for 3 days and 6 days, statistically significant increase in the cell proliferation could be observed on the cp-Ti and SLA-Ti preserved in 100 mM VitC -containing saline storage solution compared to both air group. The results revealed that the titanium surface preserved in 100 mM VitC-containing saline storage solution offered more favorable environment for cell proliferation.

### Western blotting

**Fig. 10:**
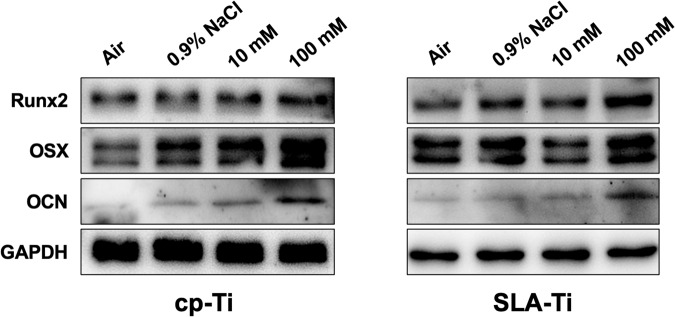
Osteogenic-related protein expression levels of Runx2, OSX, and OCN of MC3T3-E1 cells on two surfaces were detected by western blotting after culturing for 7 days

Figure 10 shows the results of the expression of osteogenesis-related proteins Runx2, Osterix, and OCN were used to evaluate the osteogenic differentiation capacity of the cells cultured on cp-Ti and SLA-Ti after being preserved in different conditions for 2 weeks. It was observed that cells cultured on cp-Ti and SLA-Ti preserved in 100 mM VitC-containing saline exhibited better osteogenic differentiation ability, and the expression of Runx2, Osterix, and OCN was higher than air, 0.9%NaCl and 10 mM VitC group.

## Discussion

Titanium implants are known for their good osseointegration properties, attributed to their biocompatibility and stability. However, there is increase attention on the aging phenomenon of titanium implants. In this study, we extained the effects of VitC-containing saline on the surface characteristics and in vitro biocompatibility of cp-Ti and SLA-Ti preserved for 2 weeks. The study results clearly revealed that titanium preserved in solution reduced hydrocarbon contamination and increased hydrophilicity. Samples preserved in 100 mM VitC-containing saline showed significantly enhancement in osteoblast adhesion, proliferation, and differentiation. Therefore, preserving titanium surfaces in a specific concentration of VitC-containing saline storage solution could reduce carbon contamination and form a favorable microenvironment for osteoblasts.

In our study, The SEM results indicated that after 2 weeks of storage, the microstructure of cp-Ti and SLA-Ti did not change significantly apart from the fact that different numbers of sodium chloride crystals and VitC crystals were observed on the surfaces of titanium stored in saline (Fig. 1). This suggests that different storage conditions and the concentration of the VitC-containing saline storage solution do not affect the inherent morphology of the titanium surface, which is corresponded with some previous research [18, 20]. However, Wennerberg et al. found that titanium plates undergo acid etching when stored in an aqueous solution, leading to the reorganization of the outermost titanium oxide layer into well defined nanostructures [21]. The findings suggested that aqueous solution seems to be better than storage using a gas barrier. Furthermore, since the freshly prepared titanium surface in liquid carries a positive charge, Na^+^ and Cl^−^ -ions in solution can be adsorbed onto the titanium surface in different bound forms [4]. As water-soluble VitC dissolves in the salt solution, the two adjacent enol hydroxyl groups at the 2nd and 3rd positions of the VitC molecule are highly susceptible to dissociation and release of H^+^, forming a hydrated ionic layer that is adsorbed onto the titanium surface by electrostatic interaction [22–25]. The Raman test demonstrated the existence of VitC crystals on titanium surfaces after 2 weeks’ storage in VitC-containing saline (Fig. 2).

**Fig. 11:**
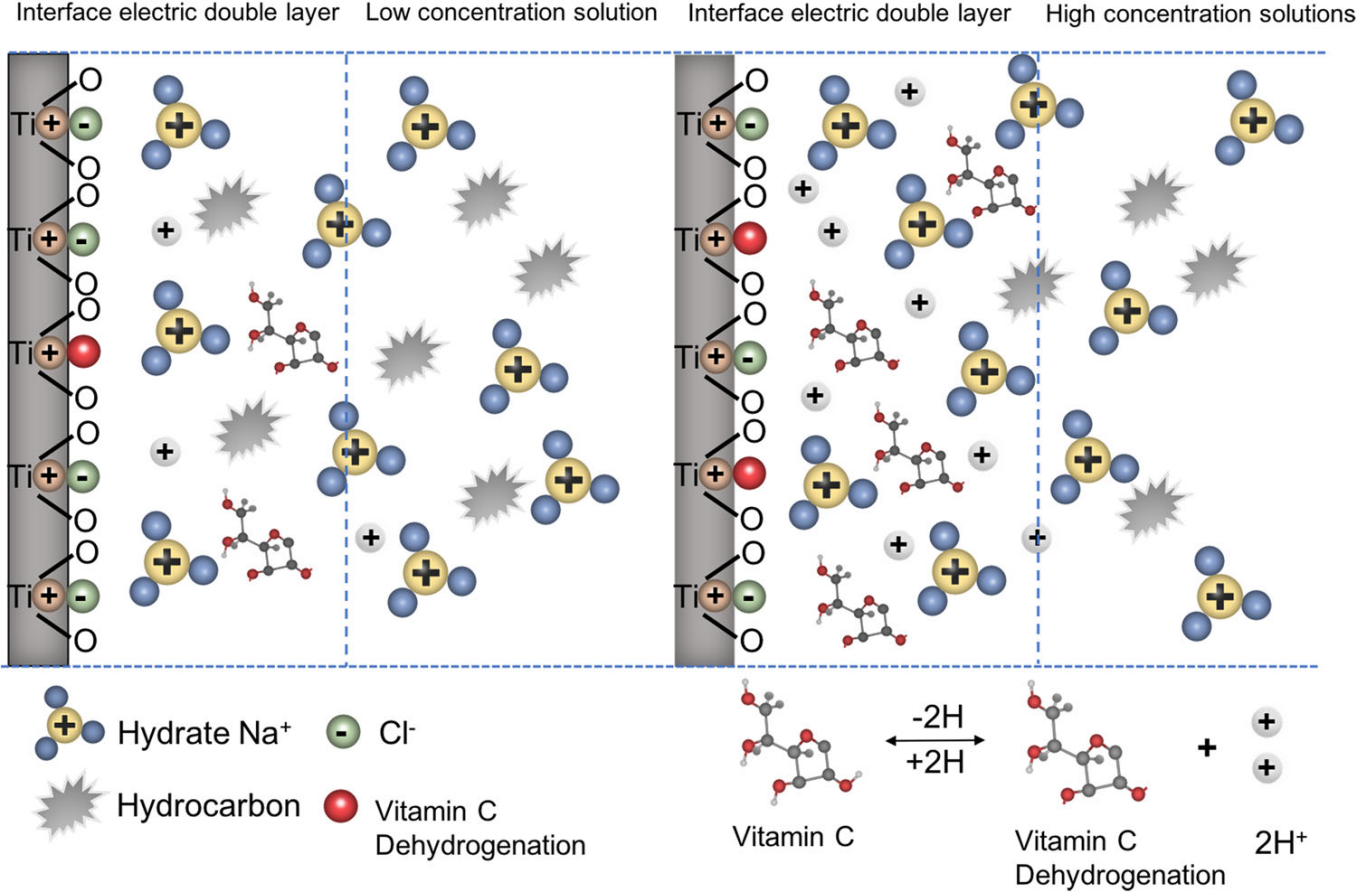
Schematic diagram of VitC-containing saline storage solution concentration on carbon accumulation in titanium surface

Hydrophilicity, which is an important characteristic affecting the biocompatibility of the titanium surface and promoting early osseointegration of implants [26]. Carbon pollution on titanium surface will reduce the hydrophilicity of titanium surface [4]. It was found that cp-Ti and SLA-Ti stored in VitC-containing saline storage solution showed eminent hydrophilicity after 2 weeks of storage. XPS analysis of the atomic composition of titanium surfaces showed that different storage conditions would cause changes in C1s of various chemical states on the titanium surface. The carbon contamination adsorbed on the surface of titanium not only exists in the form of C-C/C-H bonds, but also includes C-O bonds and C=O bonds. This is likely due to the titanium surfaces to readily adsorbing organic components from the ambient atmosphere during stoage [27]. The carbon content was relatively low on the titanium surface stored in the liquid condition, and the titanium surfaces stored in the 100 mM VitC-containing saline was the lowest (Tables 1, 2 and Figs. 3 and 4). This may be related to the competitive adsorption of storage solution solutes and hydrocarbons on titanium surfaces. As the concentration of the storage solution increases, the density of the hydrated ion layer increases after the dissociation of the enol hydroxyl groups of the aqueous sodium ions and VitC molecules of the bilayer while H ^+^ is liberated, and hydrocarbons are difficult to pass through and are sequestered outside the bilayer, thus reducing carbon accumulation on the titanium surface (Fig. 11). Roughness is an important characteristic that affects the surface morphology, cell response and early protein adsorption [28, 29]. When the cp-Ti surfaces were exposed to air, the roughness of the titanium surfaces increased compared to those stored in the 0.9% NaCl group. This increase may be attributed to carbon contamination on the titanium surfaces (Fig. 6). In addition, the roughness of the cp-Ti and SLA titanium surfaces increased with the increase of VitC concentration, which may be related to the VitC crystals adsorbed onto the titanium surfaces. These results indicated that VitC-containing saline storage solution could effectively reduce the carbon accumulation and maintain good hydrophilicity on different titanium surface (Fig. 5).

According to the previous study, after 4 weeks of storage of fresh titanium surfaces in the atmosphere, the surface carbon atoms increased from less than 20% to about 60% [30]. And carbon contamination will gradually deteriorate, which will subsequently affect early protein adsorption and cell adhesion [31]. And two independent studies found that ions adsorbed on the titanium surface are positively significant for early protein adsorption [32, 33]. We found that cp-Ti and SLA-Ti stored in 100 mM VitC-containing saline storage solution exhibited higher potential for protein adsorption (Fig. 7). Subsequently, the effect of preservation with VitC-containing saline storage solution on osteoblast behavior at different titanium surfaces was explored by cell experiments. In this study, we found that compare with titanium surfaces preserved in 0.9% NaCl solution, titanium surfaces preserved in 100 mM VitC-containing saline was able to increase more adherent osteoblasts, with more pseudopodia extending well and a larger spread area by enhancing the proteins adsorption capacity (Fig. 8). Therefore, osteoblast proliferation activity was higher in 100 mM VitC group relative to the air group and 0.9% NaCl group (Fig. 9). It is illustrated that VitC and NaCl molecules adsorbed on titanium surface can affect osteoblast adhesion and proliferation on titanium surface.

The expression of osteoblast related proteins Runx2, OSX, and OCN increased for cells cultured on both titanium surfaces in the 100 mM group (Fig. 10). These positive results may be due to the excellent hydrophilicity and biocompatibility of different titanium surfaces after storage in salt solutions with suitable concentrations of VitC. In addition, both Runx2 and Osterix are osteogenic markers with well-characterized role in the early stage of bone formation [34, 35]. OCN, which involved in the deposition of extracellular matrix, is regarded as a late marker of osteoblast differentiation [36]. The above results suggested that the VitC-containing saline storage solution group, particularly the group of 100 mM VitC-containing saline storage solution group, had superior biological activity to boost the differentiation of osteoblasts. Zhou et al. found that VitC alleviated the inhibition of osteogenesis under oxidative stress conditions [11]. Another study found that VitC was loaded onto plasma-sprayed HAP-coated commercial pure titanium (cp-Ti) surfaces and VitC-loaded HAP-coated pure titanium surfaces had good osteogenic and chemopreventive properties [36, 37]. In the 10 mM VitC group, although there has positive effect on osteoblasts, it was not as obvious as that in the 100 mM VitC group, which indicated that the suitable VitC concentration of the saline storage solution could effectively promote osteoblast adhesion, proliferation, and differentiation. Based on these research results, we have obtained a national invention patent (Patent number: ZL202010251242.3) for the application of a titanium implant storage solution containing vitamin C.

In this study, we found that after titanium surfaces with different morphologies were preserved by 100 mM VitC-containing saline storage solution, it could effectively maintain the good hydrophilicity of titanium surfaces and promote osteoblast differentiation. This also suggests that the inclusion of bioactive components in the storage solution while resisting implant aging may be beneficial to improve the bioactivity of different morphological titanium surfaces and has good application prospects. The role of hydrocarbon contamination in implant aging has been well established [37, 38]. Therefore, implants must undergo some treatments, such as ultraviolet radiation, to reduce hydrocarbon contamination before preservation [38, 39]. In addition, studies have found that some methods can be taken to reverse titanium aging before implant placement. Henningsen et al. found that non-thermal plasma treatment could activate titanium surfaces and improve the survival conditions of mouse osteoblasts [40]. Another study treated a preserved titanium surface with NaOCl for 24 h and found that NaOCl treatment essentially converted the titanium surface to a superhydrophilic state, resulting in an increased number of attached cells and enhanced cell spreading on the titanium surface [41]. It is foreseeable that preserving titanium implants with different surfaces in appropriate concentrations of storage solutions before clinical application could be an effective anti-aging strategy.

## Conclusions

The use of a VitC-containing saline solution to store cp-Ti and SLA-Ti specimens could enhance hydrophilicity of titanium surfaces and promote cell adhesion, proliferation, and osteogenic differentiation of osteoblasts. The preservation of titanium implants in a VitC-containing saline, especially the 100 mM solution, might provide a certain anti-ageing effect, which could be a useful protocol before the clinical application of titanium implants.
